# Moderate Depression Promotes Posttraumatic Growth (Ptg): A Young Population Survey 2 Years after the 2009 L’Aquila Earthquake

**DOI:** 10.2174/1745017901713010010

**Published:** 2017-03-16

**Authors:** V. Bianchini, L. Giusti, A Salza, V. Cofini, M. G. Cifone, M. Casacchia, L. Fabiani, R. Roncone

**Affiliations:** 1 Department of Mental Health, Asl Roma 5, University of L'Aquila, L'Aquila, Italy; 2 Department of Life, Health and Environmental Sciences, University of L'Aquila, L'Aquila, Italy; 3 University Rehabilitative Treatment, Early Intervention Unit, TRIP, San Salvatore Hospital, L’Aquila, Italy

**Keywords:** Anxiety, Depression, Disaster, Earthquake, PTSD, Post Traumatic Growth

## Abstract

**Background:**

Earthquakes can result in a range of psychopathology and in negative and positive consequences for survivors.

**Objective:**

To examine the association between clinical aftereffects (anxiety and depressive symptoms) and post-traumatic growth (PTG) among young survivors of the 2009 L’Aquila earthquake, Italy.

**Method:**

316 young earthquake survivors enrolled in the University of L’Aquila were evaluated two years after the natural disaster. Participants completed three main questionnaires, including Patient Health Questionnaire-9 items (PHQ-9), Self-Rating Anxiety Scale (SAS), and Posttraumatic Growth Inventory (PTGI).

**Results:**

59.6% of the student sample showed different levels of depression, whereas 13.3% reported anxiety symptoms. In both clinical dimensions (anxiety and depression), gender differences were found: female gender was confirmed risk factor for a clinical post-traumatic response. Personal PTG, demonstrated by 18% of the L’Aquila youths included in our sample, was predicted by moderate levels of depression (O.R. 2.7). In our model, gender, age, and anxiety did not show any predictive value.

**Conclusion:**

In a post-traumatic setting, the development of individual cognitive strategies is crucial, whereas after a natural disaster, paradoxically, a moderate depressive condition and the related distress could promote the drive to overcome the psychological consequences of the traumatic event.

## INTRODUCTION

The L’Aquila earthquake struck the central area of Italy on April 6, 2009. It was one of the largest natural disasters recorded in Italy in the last century, measuring 6.3 on the Richter scale. The earthquake killed 309 people, injuring over 1,600 residents. 66,000 inhabitants were displaced. Approximately 44,000 found accommodation in tent camps close to place of residence, and a further 20,000 were housed in hotels on the Abruzzo Adriatic Sea coast. Other people stayed with friends and relatives throughout Italy. Moreover, villages around the L’Aquila area were severely damaged [1].

Earthquakes are devastating, life-threatening and uncontrollable natural disasters [2]. Survivors of similar disasters experience physical and psychological distress, and several previous studies have shown that post-traumatic stress disorder (PTSD), anxiety, depression, stress, substance use disorder (SUD), and other mental health problems increase after such natural disasters [3-6].

Longitudinal studies have reported that there is a progressive decrease in symptomatology over the course of time [7, 8] and many studies have focused on factors that may be predictive for the development of psychological aftermaths [2]. Being female, having several lifetime stressors and trauma, and adopting negative coping strategies have all been shown to be predictive of psychopathological symptoms [9-11]. In addition, several aspects of earthquake experiences (*i.e.*, direct exposure, injury, close ones’ exposure, fear for the safety of close ones, persistent fear of aftershocks, prior exposure to trauma, displacement, dislocation, and house damage) represent also important factors for the development and maintenance of PTSD, anxiety, and depressive symptoms [12, 13].

Post-traumatic stress disorder (PTSD) is usually considered the most prevalent psychopathology in young people directly exposed to a deadly earthquake [14]. Casacchia *et al.* (2012) reported that the 6-month prevalence rate of PTSD among L’Aquila survivors ranged from 12% to 37.5% in different population samples (clinical and not-clinical sample [1].

Catastrophic earthquakes also have long-term psychological consequences. Giannopoulou *et al.* (2006) reported that the prevalence rate of PTSD one year after the 1999 Athens earthquake was 35.7% among youth [15]. Three years after the 1999 Parnitha earthquake in Greece, Goenjian and colleagues (2011) found that 13.6% of young people still met the criteria for clinical depression, whereas a study three years after the August 1999 earthquake in Turkey reported that the estimated rates of PTSD and comorbid depression were 40% and 18%, respectively [8, 11]. A study with young survivors reported 32% PTSD prevalence six years after the Wenchuan earthquake; among college students in the most seriously affected area, the prevalence was 14.1% [16, 2].

In fact, in addition to PTSD prevalence, several studies have reported that adults and young people exposed to traumatic events usually experience comorbid depressive and anxiety symptoms [17, 18]. Seven months after the earthquake in Canterbury, New Zealand, approximately 10% of medical students experienced moderate to extreme psychological difficulties (depression = 12%; anxiety = 9%; stress = 10%) [19].

However, traumatic experiences do not necessarily lead to the development of psychopathological symptoms and signs [14]. As such, in recent years, considerable attention has been paid to individual resilience and coping strategies adopted following trauma; in the last two decades that systematic studies have been undertaken and scales have been developed, creating the term “posttraumatic growth” (or PTG) to describe this new concept [20].

Post-traumatic growth (PTG) is characterized by subjective, adaptive psychological changes resulting from major lifetime crises or trauma. Increases in appreciation of life, personal resilience, the quality of intimate relationships, and spiritual wellbeing, as well as the resetting of life priorities and openness to new possibilities, are typical of these positive psychological changes. In PTG, positive coping strategies in the aftermath of a traumatic event occurs when an individual’s perceptions of the self, others, and the meaning of the event are positively reconstructed.

Several studies of PTG have found positive individual changes in five domains: (1) emergence of new individual opportunities and possibilities; (2) deeper relationships and greater compassion for others; (3) feeling strengthened to meet future life challenges and projects; (4) reordered priorities and fuller appreciation of life; and (5) deepening spirituality [21]. Some authors theorize that PTG is a coping style, while others think of PTG as an outcome of coping with traumatic stress. Several researchers reported that PTG can be both a coping profile and a coping outcome [22]. Our study with 411 L’Aquila college students reported low positive coping strategies and only 17.3% of the sample demonstrated PTG and positive attitudes after the earthquake traumatic experience, while the majority of sample (43.8%) showed a marked increase in substance use in the post-earthquake period [23, 24].

Instead, Jin and colleagues (2014) one year after the Wenchuan earthquake, China, involved a total of 2,300 individuals in a survey with 2,080 completing the questionnaire with a response rate of 90.4%. The PTSD prevalence estimate in this study was found to be 40.1%, and the prevalence for PTG among the participants was measured at 51.1% [25].

Several studies have analyzed the relationship between PTG and clinical post-traumatic aftereffects. On one hand, a number of studies have found that growth is related to greater well-being, less distress, and fewer posttraumatic factors [26], such as increased personal resources following trauma [27], greater self-esteem [28], and more positive mood [29], as well as less anxiety and depression [27].

On the other hand, some studies have not found any correlation between depression, anxiety, and PTG [30]. Moreover, there is evidence that greater levels of PTG may also be associated to negative affect, more distress over time, and poorer quality of life [31]. Butler *et al.* (2005) found that PTG reported in the first months after the September 11 terrorist attacks was associated with higher post trauma symptom levels [32].

In a sample of young survivors following an earthquake experience in China, the authors found that growth is a response to distress experienced. PTSD and PTG can coexist in individuals after a traumatic experience since PTSD can trigger a series of coping styles as the individual struggles with the negative outcomes after a trauma [33].

Growth and posttraumatic symptoms are not mutually exclusive. In fact, it is natural that they somehow coexist, as they share exposure to a traumatic situation as a common factor. There are indicators of how the long-term outcome of people who develop PTG differ from those who do not.

We aimed to evaluate anxiety and depressive symptomatology among a young sample of university students exposed to the L’Aquila earthquake and correlations between clinical aftereffects and individual coping strategies, specifically positive responses, such as post-traumatic growth (PTG). Our hypothesis was that PTG predicted by levels of anxiety and depression.

## MATERIALS AND METHODS

### Participants and Procedure

This study was part of a larger investigation of psychological impact among young survivors of the L’Aquila earthquake, supported by the Italian Government: the SPES PROJECT (*Supporto Psicosociale e tutela della Salute mentale nell’Emergenza Sisma), * directed by Prof. Massimo Casacchia of L’Aquila University. Data for the present study were collected two years after the L’Aquila natural disaster [1].

We only considered inclusion criteria: exposure (presence in the earthquake area) to the L’Aquila 2009 earthquake. In addition, we adopted two exclusion criteria: a major psychiatric diagnosis present at the moment of the assessment and current use of psychotropic medications.

This project was approved by the National Health Minister. Students were informed with a description of the research being conducted and that participation was voluntary and that they had a right to decline participation in the survey. Written informed consent was obtained from enrolled university students and everyone completed the study’s main questionnaires.

Three hundred and sixteen university students directly exposed to the L’Aquila earthquake agreed to participate in psychological post-disaster screening and were enrolled.

### Measures

The **ZUNG SELF RATING SCALE (SAS)** (1971) measures the anxiety-related symptoms in the physicians which was developed in 1971 to assess the severity of anxiety. The SAS questionnaire includes 20 items, with each item scored on a 4-point scale (1, never or rarely; 2, some of the time; 3, frequently; and 4, most of the time). Fifteen questions involve the assessment of increasing anxiety levels and five questions involve decreasing anxiety levels. The minimum raw score is 20, and the maximum raw score is 80; the integer part is retained in order to generate the index score (range, 25–100). Also, the scores were used to define four categories of anxiety severity: within the normal range or no significant psychopathology (20–44 points); presence of mild to moderate anxiety levels (45–59 points); severe anxiety levels (60–74 points); and presence of extreme anxiety (75–80 points). We defined anxiety symptoms to be indicated by a total index score ≥50, according to the Italian norm; it has good validity and reliability (Cronbach’s alpha = 0.85) [34].

The **PERSONAL HEALTH QUESTIONNAIRE-9 ITEMS (PHQ-9)** consists of nine items, each of which is scored from 0 to 3, providing a severity score ranging from 0 to 27. PHQ-9 severity is calculated by assigning scores of 0, 1, 2, or 3, to the response categories: not at all, several days, more than half the days, and nearly every day, respectively. The PHQ-9 total score for the nine items ranged from 0 to 27. It consists of the nine criteria for depression from the Diagnostic and Statistical Manual of Mental Disorders, fourth edition (DSM-IV). The PHQ-9 psychometric characteristics are comparable or superior and the scale is valid as both a diagnostic and severity measure. Scores used to define the PHQ-9 as a severity measure were no depression (1–4), mild depression (5–9), moderate depression (10–14), moderately severe depression (15–19), and severe depression (20–27) [35, 36].

The **POST-TRAUMATIC GROWTH INVENTORY (PTGI)** was used to measure PTG. The 21-item scale includes items that assess the degree to which an individual reports specific positive changes attributed to the struggle with a highly stressful event (possible total scores range from 0 to 105). Five empirically derived domains are assessed: *Relating to Others* (7 items) (“I have a greater sense of closeness with others”); *New Possibilities* (5 items) (“I established a new path for my life”); *Personal Strength* (4 items) (“I discovered that I am stronger than I thought I was”); *Spiritual Change* (2 items) (“I have a better understanding of spiritual matters); and *Appreciation of Life* (3 items) (“I have a greater appreciation for the values of my life”). The inventory has acceptable construct validity, internal consistency (0.90), and test-retest reliability over a 2-month interval (0.71). The PTGI consists of five subscales: Relating to others (seven items), New Possibilities (five items), Personal Strength (four items), Spiritual Change (two items), and Appreciation of Life (three items). The inventory had acceptable internal consistency (0.90) and test-retest reliability over a week interval (0.94) [27]. According to Jin *et al.* (2014) [25], PTGI scores equal to or above 57 were considered to indicate a moderate level of post-traumatic growth.

### Statistical Analysis

Descriptive statistics (frequencies and percentages) were used to study sample’s features, anxiety and depressive symptomatology, and PTG response among young survivors. A univariate ANOVA was conducted to compare gender differences in psychological scores. A Pearson correlational analysis was conducted to identify the relationships between age and psychological variables with the PTG and its sub-dimensions.

The prevalence of post-traumatic growth with a 95% CI was estimated using the categorical variable (yes = PTGI score ≥ 57, no = PTG score < 57); anxiety prevalence was calculated with the categorical variable (yes = SAS score ≥ 45, no = SAS score ranging from 20 to 44), since only 0.9% of the sample reported a SAS score ≥ 60. Depression prevalence was calculated using the categories “mild”, “moderate”, “moderately severe”, and “severe depression”, according to the PHQ-9 cut-off scores. A logistic regression analysis was performed to verify if PTG was associated with gender, anxiety, and depression.

All data were registered electronically and statistical analyses were carried out using STATA 12 software. All tests were two-tailed and p-values ≤ 0.05 were considered statistically significant.

## RESULTS

Table **1** shows the socio-demographic characteristics and the clinical evaluations of the total sample. 171 subjects (54.1%) were males and 125 (39.4%) were residents in the earthquake area. The mean age of the total sample was 24.27 ± 2.9 years. No statistical difference in age was found between genders.

Regarding anxiety symptoms, among the 314 young survivors (two missing data) the mean total SAS score was 34.62 (± 8.49), underlining the “absence of psychopathological anxiety level”. However, of the 13.3% (n = 42) (95% CI: 10.0–17.2%) who reported a significant level of anxiety (range ≥ 45); only three subjects (0.9%) demonstrated severe anxiety (range > 60). The mean PHQ-9 total score was 7.28 (± 4.6), indicating “moderate depression” levels among the study’s sample: more than 40% of the sample did not demonstrate any depressive symptoms, while 16.8% of survivors displayed “severe” depressive symptoms, and 6.2% showed “very severe depressive symptoms”. More than 18% of the students (95% CI: 14.5%–23.2%) in our sample were considered to have PTG (PTGI score ≥ 57); 56%, or 33 of these 59 students were women. Increases in *Personal Strength* (39.1%), a better *Appreciation of Life* (34.3%), and the sense of having *New Possibilities* after such a traumatic experience (25.2%) were reported in Table **1**.

The ANOVA analysis indicated a statistically significant difference for the assessed variables (anxiety level, depression symptoms, and PTG response) by gender, with higher scores for women. Significant differences were found in the following PTG subscales: *Relating to Others* (F(1, 314): 6.7; p = 0.010), *Spiritual Change* (F(1, 314): 5.34; p = 0.0215), and *Appreciation of Life* (F(1, 314): 16.40; p = 0.001) (Table **2**). No statistically significant differences were found in the dimensions of *Personal Strength* (F(1, 314): 3.56; p = 0.0602) and *New Possibilities* (F(1, 314): 3.31; p = 0.070).

Pearson coefficients were calculated to examine the correlations between total PTGI and subscale scores, and SAS and PHQ-9 total scores. Post-traumatic growth (PTGI score) and its dimensions demonstrated positive and significant correlations with all the studied clinical variables, both depression (PHQ-9 total score) and anxiety (SAS total score) levels, and with age as shown in Table **3**.

The logistic regression model, performed to evaluate the associations between the PTG responders (PTGI ≥ 57), the socio-demographic features, and the total SAS and PHQ-9 scores, indicated no associations between the characteristics of post traumatic growth, gender, and anxiety symptoms; instead the analysis reported a higher “risk”, signifying opportunity, of cultivating PTG among subjects with moderate depression (PHQ-9: 11–15) (Table **4**).

## DISCUSSION

Our study on the relationship between PTG with anxiety and depressive symptomatology among a university student community directly exposed to the 2009 L’Aquila earthquake, 2 years after the traumatic experience, showed that 13.3% of our student sample reported anxiety and close to 60% demonstrated different levels of depression, whereas symptoms of a moderate depression were predictive of PTG.

In line with previous studies [37], our results showed gender differences in both evaluated clinical dimensions (anxiety and depression): being female is confirmed as a risk factor for a clinical post- traumatic response. It is also possible that coping strategies and the use of social support differ between genders.

Our results showed low levels of anxiety: the majority of the sample reported an “absence of anxiety symptoms”, in line with previous post-disaster studies among student communities: Carter *et al.* [19] reported anxiety in 9% of young survivors 7 months after a natural disaster, and our results reported that 13% of young people experienced significant anxiety symptoms.

In terms of depression, symptoms were higher than in another international study: 2 years after the earthquake, 23% of our students showed moderate to severe depressive symptoms versus the 14% reported in a previous study conducted 3 months after the 1999 Parnitha earthquake in Greece [18].

These findings could be explained by the latency of the 2-year period between the traumatic event and the assessment conducted in our study. In fact, in a peri-traumatic setting, there is a high level of anxiety and iperarousal symptoms - see Acute Stress Disorder (APA, 2000) [38] but after the first month, this symptomatology evolves (not in all cases) toward predominantly depressive symptoms regarding the awareness of the short- and long-term consequences of trauma, for both personal and collective aftereffects (*i.e.*, dislocation, loss of social support, worsening quality of life) [39, 40].

We also found that moderate depression was related to growth, shown by only 18% of L’Aquila youths, since our analysis reported the predictive value of moderate depression in the development of PTG among our students. This was a surprising and unexpected result: a psychopathological condition, with a conventionally negative interpretation, could be hypothesized as a substrate for a positive post-traumatic response; perhaps the psychopathological condition and, therefore, the greater emotional vulnerability, could implement internal and metacognitive skills, thus promoting positive and functional coping strategies, such as PTG, in a post-traumatic setting.

Our findings regarding the association between moderate depression and PTG are in contrast with other studies and a recent study confirmed lower levels of depression in individuals with high levels of PTG [41]. Conversely, other studies have not unequivocally reported associations between PTG and psychopathology after adverse events [30]. Our results are in line with previous studies showing that greater levels of growth may also be related to negative aftermaths, more distress over time and a poor quality of life [31], higher trauma symptom levels [32], and psychological adjustment [42-44].

A possible explanation for this pattern of results is the suggestion that the relationship between growth and distress may not be so linear, as most previous studies assumed, but curvilinear [32, 45]. A part of trauma survivors may simply fail to perceive the event as a crisis, and would therefore have little reason for either distress or growth. A second group may experience distress and less post traumatic growth, and a third group may experience mostly growth and less distress. Kleim and Ehlers (2009) reported a curvilinear association between PTG and depression symptom severity: survivors with no or high growth levels reported fewer symptoms than did those who reported moderate growth, showing that growth can be related to PTSD and depressive symptoms in a nonlinear way. According to Kleim and Ehler (2009), a curvilinear association between growth and distress/psychopathology may help to resolve discrepancies among studies because the relationships found may depend on the range of perceived growth (and thus, the “regions of the curve”) occupied by a studied population: this could be a focus for a future research [46].

There are four main limitations in our study. First, the sample size was small and may not be completely representative of young L’Aquila survivors because was not represented by college students. Second, we have not considered the traumatic history of subjects, particularly the number of other types of individual trauma, a known risk factor for a worse post-traumatic response [47]. Another main limitation was the lack of a control sample, but only a minor fraction of college student was not exposed to earthquake: a comparative study should be considerate in future research. Last limitation is the use of the Post Traumatic Growth Inventory (PTGI) which lacks a specific cut-off; this underlines the need for the identification of a specific threshold to describe post traumatic growth among different general and clinical populations in different countries.

In conclusion, for most of the population, the experience of trauma does not lead to positive growth, which includes an appreciation of life, spiritual improvement, and enhanced relationships with others. Based on our results, only a small subgroup of our sample, mostly female, was able to process a positive response. Individuals struggle with the negative outcomes after a trauma and this process can imply a depressive condition. Progressively, only few trauma survivors make sense of the event (“*from meaningless world to meaningful life*”) and its philosophical and existential consequences, and in turn, they improve, despite a moderate depressive condition [48].

Our study shows the importance of assessing young people subjected to individual and/or collective traumas, not only through simple tools, such as the PHQ-9, but also through the underutilized PTG, in order to detect and treat the clinical dimension, but also to implement cognitive-behavioral interventions to promote the existential growth of young people, who are particularly vulnerable after traumatic episodes. Further analysis using a mediation model to study indirect sequential pathways through depression and positive PTSD responses could better explain our clinical hypothesis.

## Figures and Tables

**Table 1:** Socio-demographic and clinical features (anxiety and depressive symptoms) of study sample.

	**N (%)**	**Mean (SD)**
**Gender**		
M	171 (54.1%)	
F	145 (45.9%)	
**Residence**		
Earthquake area	125 (39.4%)	
Not earthquake area	192 (60.6%)	
**SAS**		34.62 (±8.49)
Total score (20-80)	
Not anxiety (20-44)	273 (86.7%)	
Mild level (45-59)	39 (12.4%)	
Severe level (60-74)	3 (0.9%)	
Very severe (75-80)	0	
**PHQ-9**		
Total score (0-27)		7.28 (±4.61)
Not depressed (≤4)	39 (12.4%)	
Mild (5-9)	(>57)	
Moderate (10-14)	Very severe (20-27)	
Severe (15-19	19 (6.2%)	
Very severe (20-27)	0	
**PTGI**		
Total score		36.37 (±21.08)
Presence PTG (>57)	59 (18.6%)	
Relating to others	29 (9%)	
New Possibilities	80 (25.2%)	
Personal Strength	124 (39.1%)	
Spiritual Change	54 (17%)	
Appreciation of life	109 (34.3%)	

**Table 2:** Means, standard deviations and F test (Univariate ANOVA) for differences in SAS total score (anxiety), PHQ-9 total score (depression) and PTGI total score and its dimensions by gender.

		**Male**		**Female**		**F**	**p**
	Cronbach’s alpha	Mean	SD	Mean	SD		
**SAS total score**	0.7986	32.58	7.28	37.01	9.18	22.85	0.000*
**PHQ-9**	0.8765	6.75	4.57	7.90	4.60	4.93	0.027*
**PTGI**	0.8730	33.16	20.85	40.14	20.79	8.80	0.003*
Relating to others	0.8175	8.62	7.21	10.66	6.66	6.7	0.010*
New Possibilities	0.8130	9.39	6.75	10.74	6.24	3.31	0.070
Personal Strength	0.8341	7.40	4.97	8.46	4.90	3.56	0.060
Spiritual Change	0.8450	1.44	2.41	2.12	2.76	5.34	0.021*
Appreciation of Life	0.8281	6.46	4.10	8.26	3.74	16.73	0.001*

Note: *p<.05

**Table 3:** Correlation (*Bravais Pearson coefficient*) among the study variables (PTGI score and subscales, SAS, PHQ-9 total score, age).

	**1**	**2**	**3**	**4**	**5**	**6**	**7**	**8**	**9**	**10**
**PTGI total score**	1									
**PHQ-9 total score**	0.12*	1								
**SAS total score**	0.14*	0.66*	1							
Relating to others	0.89*	0.14*	0.13*	1						
New Possibilities	0.91*	0.16*	0.14*	0.78*	1					
Personal strength	0.82*	-0.03	0.03	0.62*	0.70*	1				
Spiritual change	0.59*	0.14*	0.20*	0.50*	0.46*	0.34*	1			
Appreciation of life	0.78*	0.09	0.15*	0.61*	0.64*	0.61*	0.43*	1		
age	0.01	-0.02	-0.05	-0.06	0.06	-0.00	-0.02	0.04	1	

Note: *p<0.05

**Table 4:** Multiple logistic model: association between the PTG responders (PTGI≥57), gender and clinical response (anxiety and depression).

Variables		OR	IC 95%
**Gender**			
	F	Rif.	0.33-1.08
	M	0.60	
**SAS score**			
	Yes (>45)	Rif.	0.24-1.64
	No (≤44)	0.63	
**PHQ-9 score**			
	No Depression (≤4)	Rif.	
	Moderate level (10-14)	Severe depression (15-19)	
	No Depression (≤4)	Mild level (5-9)	1.28
	Moderate level (10-14)	2.70	1.20-6.09
	Severe depression (15-19)	1.31	0.31-5.47
